# Concerted Action of the Ubiquitin-Fusion Degradation Protein 1 (Ufd1) and Sumo-Targeted Ubiquitin Ligases (STUbLs) in the DNA-Damage Response

**DOI:** 10.1371/journal.pone.0080442

**Published:** 2013-11-12

**Authors:** Julie Bonne Køhler, Maria Louise Mønster Jørgensen, Gabriele Beinoraité, Michael Thorsen, Geneviève Thon

**Affiliations:** Department of Biology, University of Copenhagen, Copenhagen, Denmark; University of Minnesota, United States of America

## Abstract

In eukaryotes many players in the DNA-damage response (DDR) catalyze protein sumoylation or ubiquitylation. Emphasis has been placed on how these modifications orchestrate the sequential recruitment of repair factors to sites of DNA damage or stalled replication forks. Here, we shed light on a pathway in which sumoylated factors are eliminated through the coupled action of Sumo-targeted ubiquitin ligases (STUbLs) and the ubiquitin-fusion degradation protein 1 (Ufd1). Ufd1 is a subunit of the Cdc48-Ufd1-Npl4 complex implicated in the sorting of ubiquitylated substrates for degradation by the proteasome. We find that in fission yeast, Ufd1 interacts physically and functionally with the Sumo-targeted ubiquitin ligase (STUbL) Rfp1, homologous to human RNF4, and with the Sumo E3 ligase Pli1, homologous to human PIAS1. Deleting a C-terminal domain of Ufd1 that mediates the interaction of Ufd1 with Rfp1, Pli1, and Sumo (*ufd1ΔCt* ^*213-342*^) lead to an accumulation of high-molecular-weight Sumo conjugates and caused severe genomic instabilities. The spectrum of sensitivity of *ufd1ΔCt* ^*213-342*^ cells to genotoxins, the epistatic relationships of *ufd1ΔCt* ^*213-342*^ with mutations in DNA repair factors, and the localization of the repair factor Rad22 in *ufd1ΔCt* ^*213-342*^ cells point to *ufd1ΔCt* ^*213-342*^ cells accumulating aberrant structures during replication that require homologous recombination (HR) for their repair. We present evidence that HR is however often not successful in *ufd1ΔCt* ^*213-342*^ cells and we identify Rad22 as one of the high-molecular-weight conjugates accumulating in the *ufd1ΔCt* ^*213-342*^ mutant consistent with Rad22 being a STUbL/Ufd1 substrate. Suggesting a direct role of Ufd1 in the processing of Sumo-conjugates, Ufd1 formed nuclear foci colocalizing with Sumo during the DDR, and Sumo-conjugates accumulated in foci in the *ufd1ΔCt* ^*213-342*^ mutant. Broader functional relationships between Ufd1 and STUbLs conceivably affect numerous cellular processes beyond the DDR.

## Introduction

The small modifiers SUMO and ubiquitin are effectors of many regulatory changes occurring in eukaryotic cells. In reactions catalyzed by E1, E2 and E3 enzymes that act in a cascade, SUMO or ubiquitin can each be conjugated to lysine residues of target proteins. Conjugation often results in a change in the interaction properties of the target. In contrast to the ubiquitin pathway where substrate selection is mediated by large sets of E2 and E3 enzymes, sumoylation appears restricted to the use of very few E2 and E3 enzymes in all organisms examined to date [1-3]. In fission yeast the only known SUMO E3 ligases are Nse2 and the PIAS family member Pli1. Nse2 and Pli1 are both of the SP-RING type, functioning together with a single E2 enzyme, Ubc9 (also called Hus5; [4-6]). 

Among the many processes affected by sumoylation or ubiquitylation a special focus has been on the roles played by these modifications in DNA replication and repair. Many repair enzymes catalyze ubiquitylation or sumoylation and many factors acting at stalled replication forks or other DNA lesions are conditionally ubiquitylated or sumoylated [5,7-20]. Their modification can affect downstream repair events or repair pathway choices. The molecular mechanisms through which the modifications operate are known in a few cases. One of the best-understood examples is modification of the proliferating cell nuclear antigen (PCNA) sliding-clamp by either mono- or poly-ubiquitin which signals distinct bypass strategies in the face of replication-stalling lesions [21-23]. Another extensively studied, albeit less understood, event is sumoylation of the HR factor Rad52. *S. cerevisiae* Rad52 and its fission yeast homolog Rad22 are established sumoylation targets [7,8]. Sumoylation of Rad52 has been proposed to influence the efficiency with which HR proceeds by altering protein stability and by reducing the affinity of Rad52 for DNA [8,24]. In yet another example, in mammalian cells, the RNF8 E3 ubiquitin ligase mediates DSB repair. An RNF8-initiated ubiquitylation cascade centered on lesion-flanking histones H2A and H2AX drives the sequential recruitment of repair factors in a manner partly dependent on concurrent sumoylation events [18,12,13,25]. Much emphasis is currently being placed on how cross talks between the ubiquitylation and sumoylation pathways affect repair factors and DNA transactions. 

Providing mechanistic insight for how sumoylation and ubiquitylation events might be coupled, some ubiquitin E3 ligases of the RING family can recognize sumoylated proteins *via* SUMO-interacting motifs (SIMs). These SUMO-targeted ubiquitin ligases or STUbLs were defined by the Rfp1/Slx8 and Rfp2/Slx8 dimers in *S. pombe*, the Slx5/Slx8 dimer in *S. cerevisiae* and human RNF4 [26-30]. Modification by STUbLs can lead to substrate degradation by the proteasome [30], and it might also mediate non-proteolytic functions. The impact of STUbLs on cellular SUMO homeostasis is revealed by increased sumoylation levels and accumulation of poly-SUMO chains in STUbL mutants [26-29,31]. The imbalance in SUMO dynamics in these mutants is associated with genomic instabilities and slow-growth phenotypes. Consistent with direct roles in genome maintenance, both human and *S. cerevisae* STUbLs have been seen at DSBs or sites of active replication [9,32-35]. Other classes of ubiquitin ligases might also function as STUbLs. This was shown in a recent study for the *S. cerevisiae* Rad18 ubiquitin ligase [36].

The molecular recognitions and sequence of events linking sumoylation, ubiquitylation and substrate degradation, the substrate specificities and interaction partners of STUbLs remain largely unknown. We attempted to shed light on some of these issues by searching for proteins interacting with the fission yeast SUMO E3 ligase Pli1 or the STUbL Rfp1. Ufd1 was identified in both searches. Ufd1 makes up together with Npl4 a major substrate-recruiting co-factor of the homo-hexameric Cdc48 AAA^+^ ATPase (p97 in mammals) [37], which is implicated in various ubiquitin-dependent processes in the cell [38,39]. We found that a mutant with a C-terminal truncation of Ufd1 that removes the interaction domain with Pli1 and Rfp1 (*ufd1ΔCt* ^*213-342*^) displayed many phenotypic similarities with STUbL mutants. Like STUbL mutants, *ufd1ΔCt* ^*213-342*^ cells accumulated sumoylated proteins, they were hypersensitive to DNA-damaging agents and they displayed frequent spontaneous Rad22 foci. Also, the epistatic relationship of *ufd1ΔCt* ^*213-342*^ to mutations in HR factors was the same as for STUbL mutants. Combined with localization studies of Ufd1 and SUMO in cells subjected to genotoxic stress, these phenotypes lead us to refine a proposed function for Ufd1 in genome maintenance and in the STUbL pathway. 

## Results

### Identification of physical and functional interactions between Pli1, Rfp1, and Ufd1

**Figure 1:**
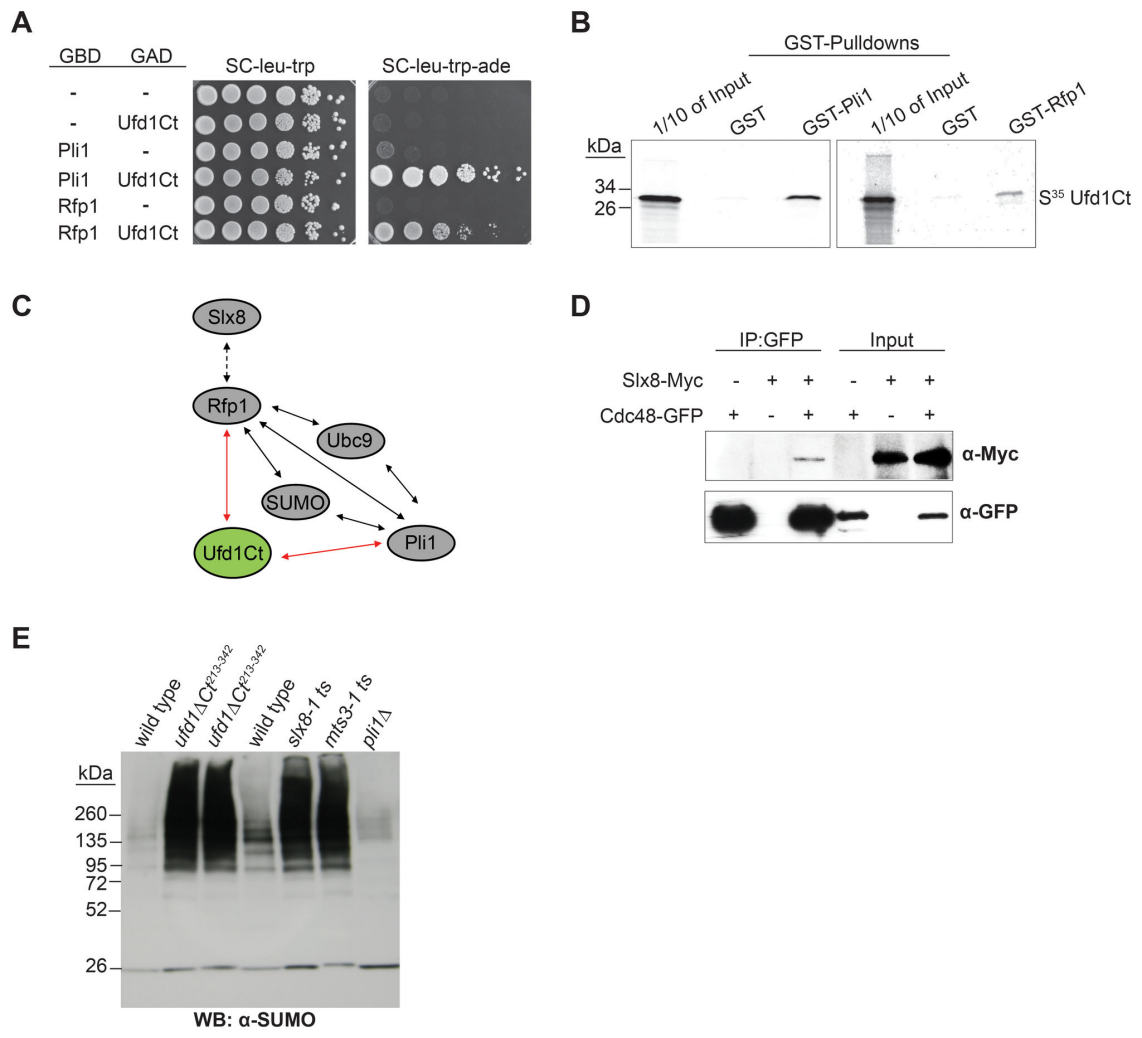
Physical and functional interactions between *S. pombe* Ufd1/Cdc48, the STUbL Rfp1/Slx8 and the SUMO E3 ligase Pli1. (A) Yeast two-hybrid interactions. 10-fold dilution series of *S*. *cerevisae* strain PJ69-4A expressing a C-terminal domain of Ufd1 (aa 248-342) fused to the Gal4 DNA activation domain (GAD) together with either Pli1 or Rfp1 fused to the Gal4 DNA-binding domain (GBD) were spotted on the indicated media. Protein interactions result in activation of the *ADE2* reporter gene in the tester strain and growth on SC-leu-trp-ade. -: empty vectors. (B) Ufd1Ct interacts with Pli1 and Rfp1 *in* *vitro*. GST pull-down experiment with GST, GST- Pli1 or GST-Rfp1 incubated with *in* *vitro*-translated, ^35^S-labeled Ufd1 (aa 110-342). (C) Summary of two-hybrid interactions. Red arrows indicate novel interactions identified in this study; black arrows, previously reported interactions that were (solid arrows) or not (dashed arrows) also identified in our screens. (D) Co-purification of Cdc48 and Slx8. Cdc48-GFP was purified on a GFP affinity matrix from cells expressing Myc-tagged Slx8 or an untagged control. Cdc48-GFP and Slx8-Myc were detected by Western blotting with respectively GFP and Myc antibodies. (E) Accumulation of sumoylated proteins in *ufd1ΔCt* ^*213-342*^ cells. Whole-cell extracts of wild type and indicated mutants were probed with an anti-SUMO antibody. The *slx8-1* and *mts3-1* mutants were shifted from 30°C to 37°C for 4 hr prior to harvesting; all other strains were propagated at 30°C. The strains were, from left to right, JK8; JK9; JK10; JK11; NBY1008; PI131; Δsph2.

We conducted large-scale two-hybrid screens using respectively the fission yeast SUMO E3 ligase Pli1 and the STUbL protein Rfp1 as baits to identify factors acting in concert with these proteins (Figure 1). An interaction between Pli1 and Rfp1 and interactions of the two proteins with sumoylation factors (SUMO and Ubc9) were observed in the screens, confirming previous reports ([26,28]; Figure 1A). Yet-undescribed interactions were also revealed by both screens. In particular, both Pli1 and Rfp1 interacted with a C-terminal domain of Ufd1 (Figure 1A). The interactions were confirmed in *in vitro* GST pull-downs (Figure 1B), indicating the proteins make direct contact with each other. 

Many reports document the role of Ufd1 in endoplasmic reticulum associated degradation (ERAD) where Ufd1 functions in complex with the Cdc48/p97 ATPase and Npl4 to sort and extract proteins destined for degradation [40,41]. Other studies have suggested broader roles for the Cdc48-Ufd1-Npl4 complex in ubiquitin-dependent processes [42,43,38,39], with a recent focus on events occurring in chromatin [44-54]. Here, the ability of Ufd1 to interact with both Pli1 and Rfp1 suggested that Ufd1 might facilitate the degradation of STUbL substrates. Further suggesting a direct physical relationship between the STUbL pathway and the Cdc48-Ufd1-Npl4 complex, Slx8 (the catalytic subunit of the Slx8/Rfp1 STUbL dimer) co-purified with Cdc48 in immunoprecipitations of Cdc48 from *S. pombe* cell extracts (Figure 1D). Together these observations motivated a further investigation of a possible functional relationship between Cdc48-Ufd1-Npl4 and STUbLs. We created a mutant lacking the C-terminal domain of Ufd1 (*ufd1ΔCt* ^*213-342*^ encoding Ufd1 with a deletion of aa 213-342) which includes the part responsible for the two-hybrid interactions of Ufd1 with Pli1 and Rfp1. According to structural data, Ufd1 binds ubiquitin *via* an N-terminal domain while the C-terminal portion of the protein interacts with Cdc48 and Npl4 [40,55-57]. Some aspects of Cdc48/Ufd1/Npl4 complex formation might be affected in *ufd1ΔCt* ^*213-342*^ cells but remaining interactions bridged by ubiquitin or other shared interaction partners possibly account for the fact that the *ufd1ΔCt* ^*213-342*^ mutant is viable in contrast to the full *ufd1* deletion [58].

As can be seen in Figure 1E, the *ufd1ΔCt* ^*213-342*^ mutant accumulated sumoylated species that migrated slowly in denaturing polyacrylamide gels. This is similar to what is observed when STUbL function is compromised ([26-28]; Figure 1E). The slowly-migrating species might be high-molecular weight, poly-modified proteins, or they might contain branched molecules whose migration would be retarded in gels. The elevated level of SUMO conjugates in *ufd1ΔCt* ^*213-342*^ cells suggests that Ufd1 normally participates in the down-regulation of these conjugates. 

### SUMO accumulates in nuclear foci when Ufd1 function is impaired

**Figure 2:**
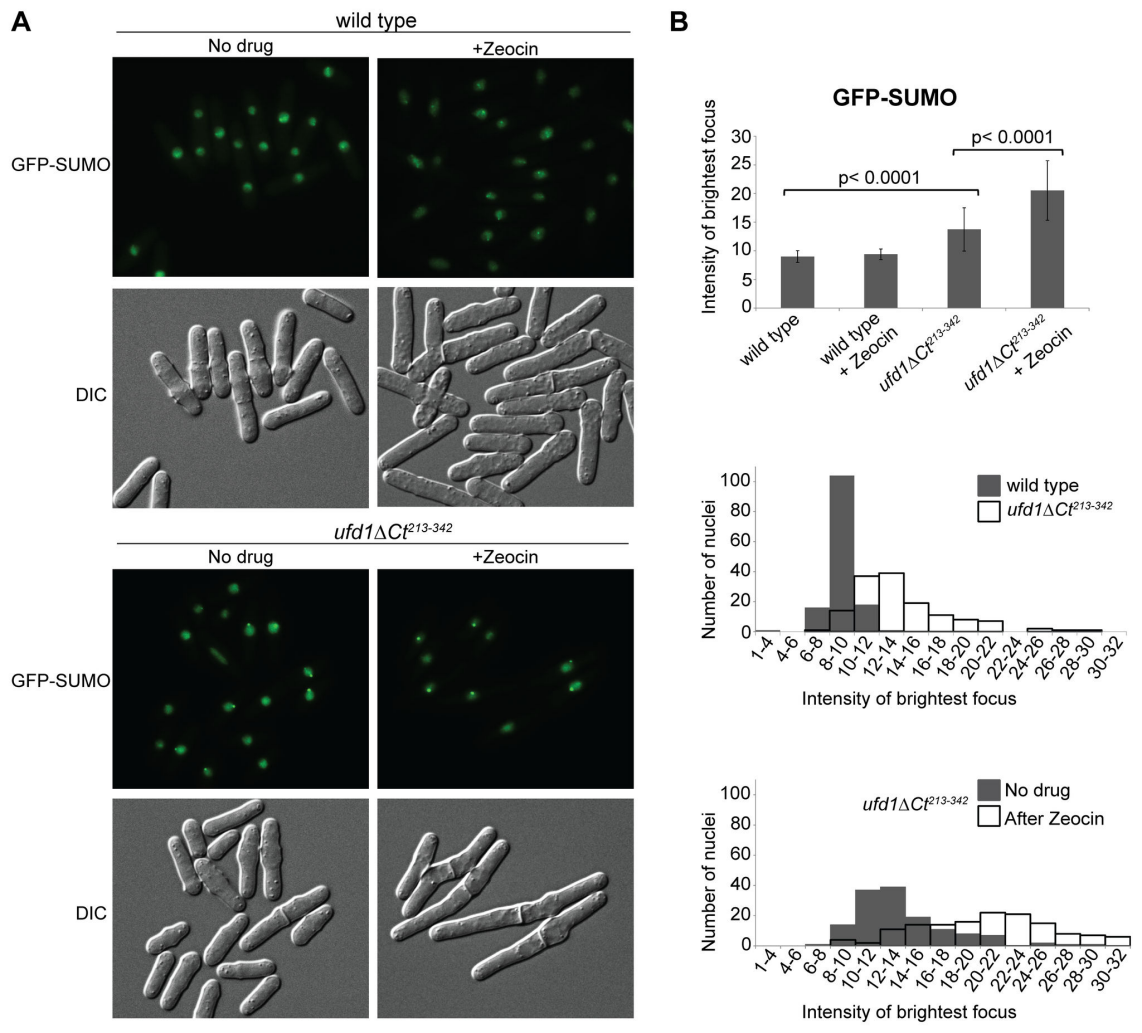
Increased SUMO foci intensity in *ufd1ΔCt* ^*213-342*^ cells. (A) Fluorescence imaging of GFP-SUMO in wild-type and *ufd1ΔCt* ^*213-342*^ cells propagated in the presence or absence of Zeocin (350 µg/ml for 2 1/2 hr). (B) Quantification of GFP-SUMO foci intensity. The intensity of the brightest GFP voxel was measured in > 140 nuclei for each of the strains and conditions shown in (A). Bar graphs in the upper panel display the mean intensities and standard deviations measured in the experiment; histograms in the two lower panels show the distribution of GFP-SUMO intensities. Statistically-significant differences (p ˂ 0.0001) between *ufd1ΔCt* ^*213-342*^ cells and wild type, and between *ufd1ΔCt* ^*213-342*^ cells before and after Zeocin treatment were observed in this experiment and for two replicates of the experiment (not shown).

We set up to determine the intracellular localization of the SUMO conjugates that accumulate in *ufd1ΔCt* ^*213-342*^ cells. In *S. pombe*, SUMO detected by immunofluorescence or GFP-tagging produces a somewhat diffuse nuclear signal, punctuated by more intense nuclear foci [59]. The most intense SUMO focus colocalizes with the clustered centromeres at the spindle-pole body [59]. In our fluorescence microscopy images, GFP-SUMO foci appeared strikingly brighter in the *ufd1ΔCt* ^*213-342*^ mutant than in wild-type cells imaged in parallel for comparison (Figure 2A). Figure 2B plots the voxels of highest intensity in *ufd1ΔCt* ^*213-342*^ and wild-type nuclei. Despite a certain degree of variation between cells, higher intensities were consistently measured in the *ufd1ΔCt* ^*213-342*^ mutant confirming simple visual inspection. Thus, the high molecular-weight SUMO-containing species detected by Western blotting in *ufd1ΔCt* ^*213-342*^ cells appear to accumulate preferentially at specific subnuclear loci. 

### Colocalization of SUMO and Ufd1 during the DNA-damage response

**Figure 3:**
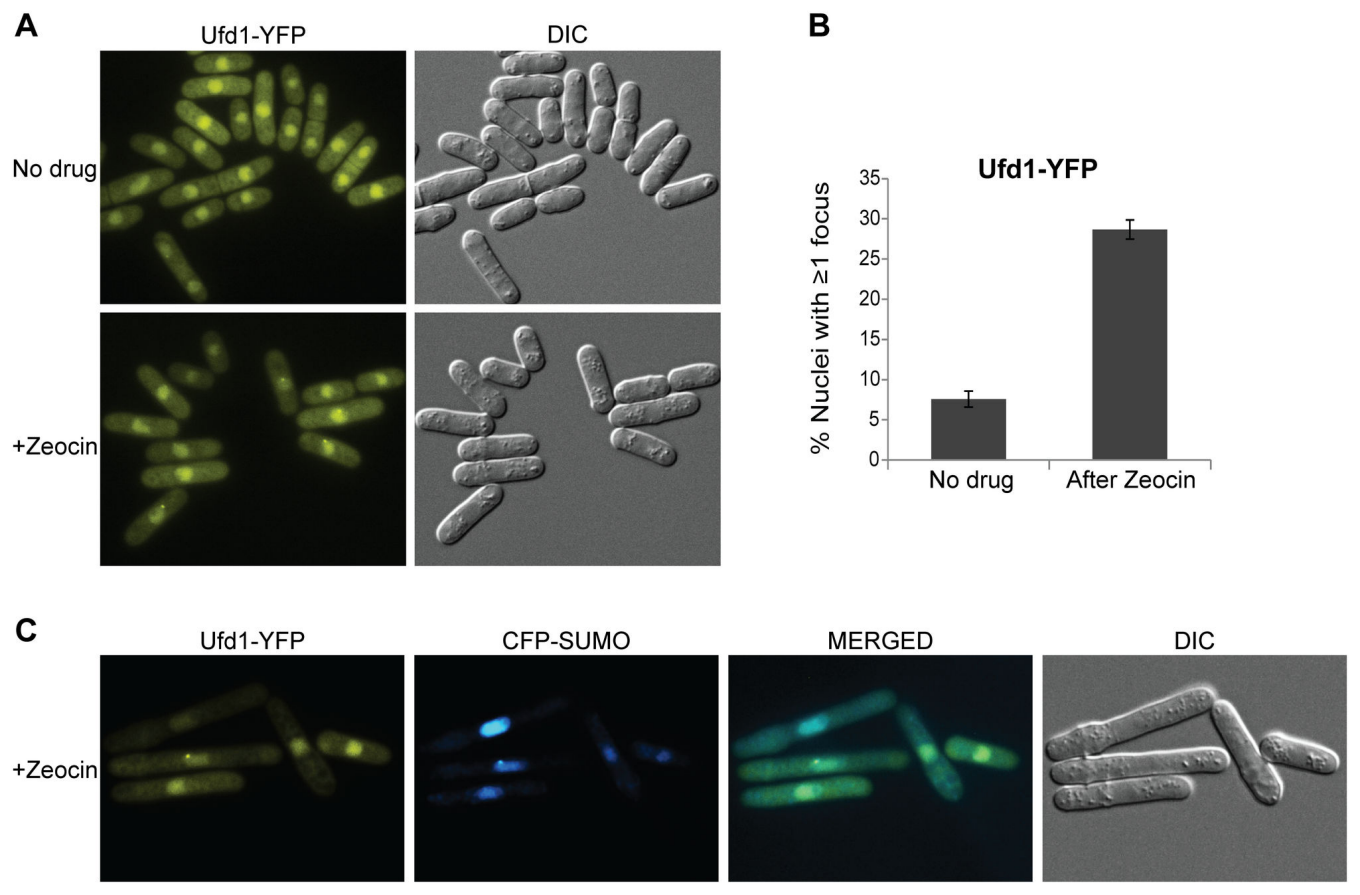
Ufd1 forms foci upon DNA damage which colocalize with CFP-SUMO. (A) Fluorescence imaging of cells expressing Ufd1-YFP propagated in the presence or absence of Zeocin (350 µg/ml for 3 1/2 hrs). (B) Bars represent the percentage of cells with at least one Ufd1-YFP focus before and after Zeocin treatment averaged from three independent experiments. (C) Colocalization of Ufd1-YFP with CFP-SUMO after Zeocin treatment.

Cdc48 and Ufd1 are both predominantly nuclear in fission yeast ([60,61]; Figure 3A). We observed that a Ufd1-YFP fusion protein formed nuclear foci at low frequency in a wild-type background. The DSB-inducing drug Zeocin increased the frequency of Ufd1-YFP foci so that approximately 30% of cells displayed at least one focus after 3-4 hrs of exposure to Zeocin (Figure 3B). In the vast majority of cases, a single focus was detected. This focus was at the nuclear periphery and colocalized with the most intense CFP-SUMO spot in the same nuclei (Figure 3C). Colocalization of Ufd1 and SUMO indicates that Ufd1 participates in the turn-over of SUMO-conjugates in a direct manner. Ufd1-YFP foci would be induced by increased sumoylation activities occurring as part of the DNA damage response whose products need to be turned-over by Ufd1. Consistent with a need for Ufd1 for this turn-over, GFP-SUMO foci increased in intensity in the *ufd1ΔCt* ^*213-342*^ background after Zeocin treatment (Figure 2B). GFP-SUMO foci did not increase in intensity in wild-type cells after Zeocin treatment (Figure 2B) indicating that sumoylation events induced by DNA damage are efficiently turned-over by Ufd1 in wild type. 

### Genome instability in *ufd1ΔCt* ^213-342^ mutant cells

**Figure 4:**
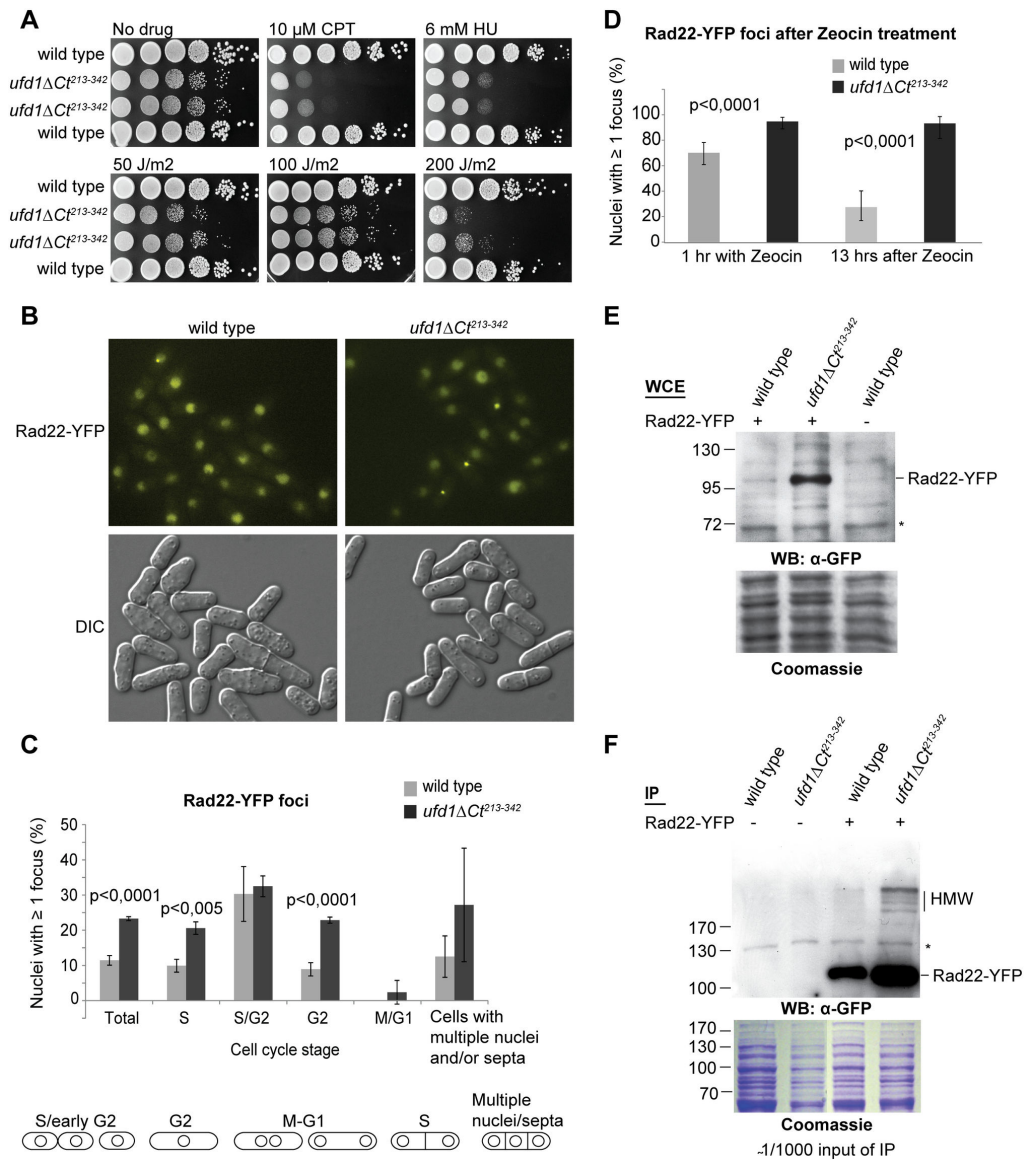
Ufd1 is required for the maintenance of genome integrity. (A) *ufd1ΔCt* ^*213-342*^ cells are sensitive to DNA damage: 10-fold dilution series of the indicated strains were spotted onto YES plates containing camptothecin (CPT) or hydroxyurea (HU), or subsequently exposed to UV irradiation as indicated. (B) Fluorescence imaging of wild-type and *ufd1ΔCt* ^*213-342*^ asynchronous cultures expressing Rad22-YFP. (C) *ufd1ΔCt* ^*213-342*^ cells display an increased frequency of spontaneous Rad22 foci: Nuclei were classified according to their position in the cell cycle based on cell morphology. Bars represent the percentage of nuclei with at least one Rad22-YFP focus averaged from three independent experiments. Error bars correspond to the standard deviations for the combined data and p-values were calculated with a Fishers exact test. More than 300 cells of each strain were counted in each experiment, the numbers are reported in Table S2. Only very few cells were counted in the ‘multiple nuclei/septa’ category, giving rise to the large standard deviations in this category. (D) *ufd1ΔCt* ^*213-342*^ cells are unable to recover from Zeocin-induced damage: Rad22-YFP foci were quantified after 1 hr of growth in Zeocin-containing medium (300 µg/ml) and again 13 hr after Zeocin had been removed from the media. Between 50 and 100 cells were counted for each strain at each time point; error bars indicate exact binomial 95% confidence intervals. Indicated p-values were obtained with a Fishers exact test. Fluorescence images are shown in Figure S3. (E) and (F) Rad22-YFP accumulation in *ufd1ΔCt* ^*213-342*^ cells: (E) Rad22-YFP from whole cell extracts of the indicated strains was detected by an anti-GFP antibody. (F) Rad22-YFP was affinity-purified from a wild-type or *ufd1ΔCt* ^*213-342*^ genetic background and detected by anti-GFP immunoblotting. HMW indicates higher molecular weight species of Rad22-YFP accumulating in *ufd1ΔCt* ^*213-342*^. * indicates a crossreacting species.

STUbL mutants are sensitive to genotoxic stress [26-28]. We tested whether the *ufd1ΔCt* ^*213-342*^ mutant might be similarly sensitive to DNA-damaging agents by exposing cells to camptothecin (CPT), hydroxyurea (HU) or UV irradiation. We found that *ufd1ΔCt* ^*213-342*^ cells were not particularly sensitive to low doses of UV irradiation (50 J/m^2^) but they were hypersensitive to higher doses as well as to respectively HU and CPT (Figure 4A). The toxic effect of CPT is thought to occur during DNA replication when incoming replication forks collide with topoisomerase 1-DNA complexes stabilized by CPT, resulting in fork collapse [62]. High doses of UV light or chronic exposure to HU can lead to similar types of damage [63,64]. Hence the spectrum of sensitivities of *ufd1ΔCt* ^*213-342*^ cells indicates that the mutant might fail to repair aberrant DNA structures that arise from replicative stress, such as collapsed replication forks and resulting double-strand breaks (DSBs). 

Consistent with the hypersensitivity of the *ufd1ΔCt* ^*213-342*^ mutant to DNA stress, we observed more spontaneous Rad22-YFP foci in *ufd1ΔCt* ^*213-342*^ cells than in wild type (Figure 4B and C). In addition, the Rad22-YFP foci often appeared more intense in the *ufd1ΔCt* ^*213-342*^ mutant. As a central player in HR, Rad22 forms foci at DSBs and other regions with exposed ssDNA where HR engages in repair [65]. Analysis of foci distribution along the cell cycle in *ufd1ΔCt* ^*213-342*^ cells found that Rad22 foci appeared during S-phase and persisted into G2. This is in contrast to the wild-type situation in which recombination-based repair of damage occurring during S-phase is delayed until the end of replication, leading to a temporary accumulation of Rad22 foci only in late S-phase/G2 cells ([66,67]; Figure 4C). The greater number of S-phase cells containing Rad22 foci thus suggest that the *ufd1ΔCt* ^*213-342*^ mutant suffers more damage during replication such as collapsed replication forks that present substrates for HR proteins. The persistence of foci during G2 further suggests that *ufd1ΔCt* ^*213-342*^ cells are also impaired in the recovery from DNA damage through HR. Consistently, *ufd1ΔCt* ^*213-342*^ cells recovered poorly from Zeocin treatment and cells arrested with bright Rad22 foci indicating incomplete HR events (Figure 4D). Thus as for STUbLs [26,28], impaired Ufd1 function decreases the resistance to various forms of genotoxic insults including replicative stress. Moreover, as for STUbL mutants [26,27], the G2/M checkpoint appeared functional in the *ufd1ΔCt* ^*213-342*^ mutant as Rad22 foci were not observed in mitosis, indicating that cells are prevented from dividing with unrepaired DNA. Furthermore, *ufd1ΔCt* ^*213-342*^ cells were in general elongated suggesting delayed cell cycle progression due to checkpoint activation and they did not display a ‘*cut*’ phenotype. 

Rad22 is a known target for sumoylation in *S. pombe* [7]. Its sumoylation is catalyzed by Pli1 [4]. We examined the modification status of Rad22 in wild-type and *ufd1ΔCt* ^*213-342*^ cells by Western blot. Corroborating our microscopy results we observed that Rad22 protein levels were increased in *ufd1ΔCt* ^*213-342*^ cells compared to wild type as seen in whole cell extracts and by pull-down using the Rad22-YFP construct (Figure 4E and F). Furthermore, immunoprecipitation of Rad22-YFP revealed that slowly-migrating forms of Rad22 were enriched in *ufd1ΔCt* ^*213-342*^ cells (Figure 4F), suggesting Rad22 is a substrate in the STUbL/Ufd1 pathway. The observed increase in Rad22 amounts in the *ufd1ΔCt* ^*213-342*^ mutant could be due either to increased protein synthesis or to protein stabilization. A recent report proposes Rad22 protein levels are controlled by a proteasomal pathway involving the proteasome-associated factor Bag101. Consistent with this the authors detected more Rad22 in proteasome mutants [68]. Yet another study, in *S. cerevisae*, found that mutations in proteasomal subunits influence Rad52 levels rather through increased transcription [69]. However, as we did not observe any change in *rad22* mRNA expression in *ufd1ΔCt* ^*213-342*^ cells as judged by quantitative PCR (Figure S3), our data suggest that Rad22 may instead accumulate in the *ufd1ΔCt* ^*213-342*^ background due to stabilization. 

### Epistasis with Pli1

**Figure 5:**
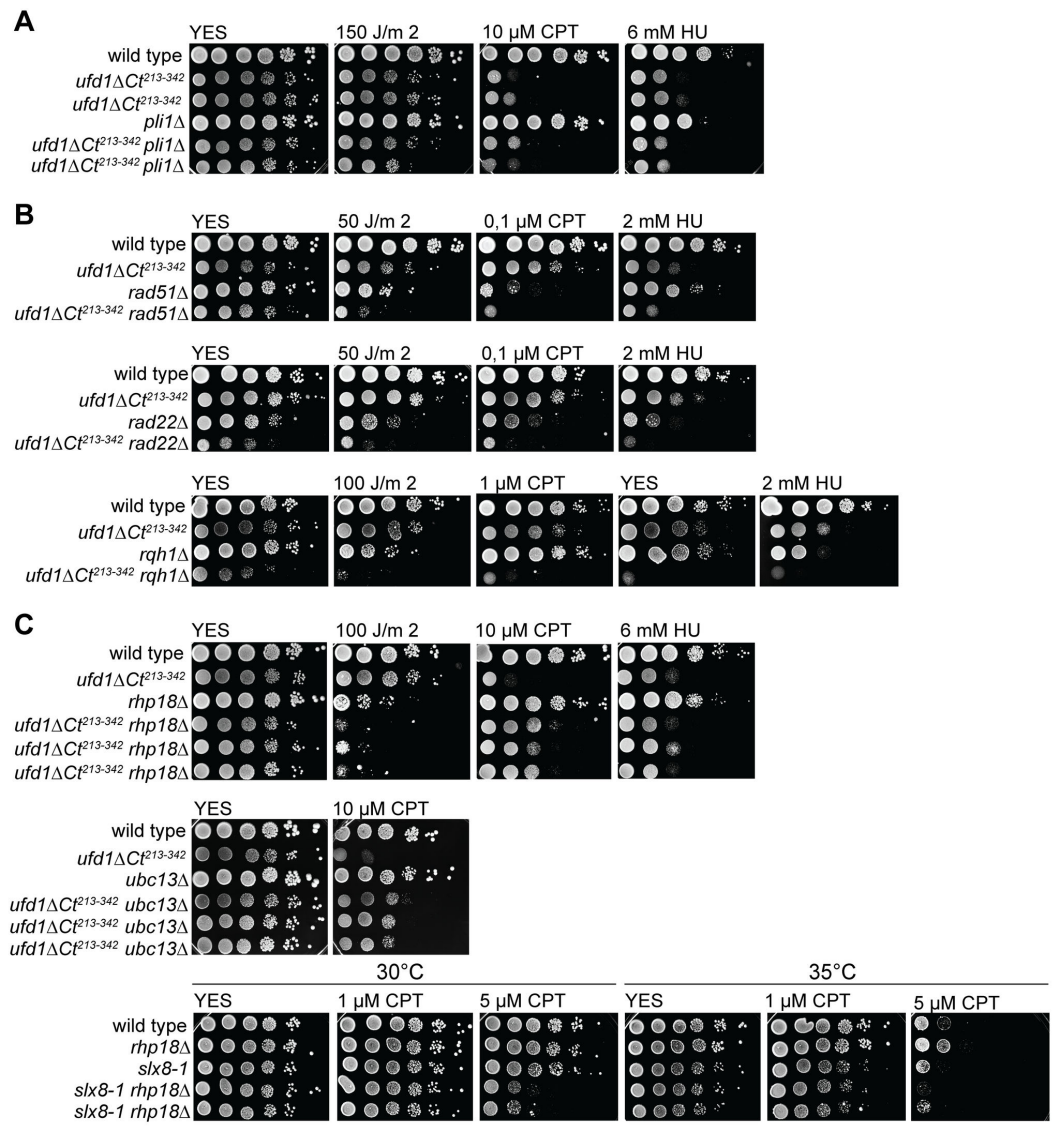
Epistasis analysis of the *ufd1ΔCt* ^*213-342*^ mutation with mutations in DNA repair or STUbL pathway. (A, B, C) 10-fold serial dilutions of the indicated strains were spotted onto YES plates with or without DNA-damaging agents as indicated, or onto plates subsequently exposed to UV irradiation. (A) Epistatic relationship of *ufd1ΔCt* ^*213-342*^ with *pli1Δ* indicates non-overlapping functions of Ufd1 and the STUbL pathway. (B) Negative genetic interactions between *ufd1ΔCt* ^*213-342*^ and mutations of HR repair proteins. (C) Partial suppression of *ufd1ΔCt* ^*213-342*^ CPT sensitivity by *rhp18Δ* and *ubc13Δ*.

Reducing SUMO-conjugate formation by either deleting the major SUMO E3 ligase Pli1 (*pli1∆*) or overexpressing the deSUMOylase Ulp2 suppresses the growth defects and genotoxin sensitivity of STUbL mutants [26,27]. In contrast, *pli1∆* failed to rescue the growth defect or damage sensitivity of *ufd1ΔCt* ^*213-342*^ cells; if anything the *ufd1ΔCt* ^*213-342*^ *pli1∆* double mutant grew slightly more poorly than the single *ufd1ΔCt* ^*213-342*^ mutant (Figure 5A). This shows that the growth impairment and DNA-damage sensitivity in *ufd1ΔCt* ^*213-342*^ cells are not merely caused by deregulation of Pli1-dependent SUMO conjugates, as inferred for STUbL mutants.

### Epistasis analysis with Rad22, Rhp51, Rqh1 and Rhp18

The relationship between Ufd1 and the DNA-damage response was investigated further through epistasis analyses. Synthetic growth defects and increased sensitivity to DNA-damaging agents were observed when the *ufd1ΔCt* ^*213-342*^ mutation was combined with the deletion of factors required for HR, respectively *rad22∆*, *rhp51∆* and *rqh1∆* (Figure 5B). These phenotypes are consistent with the *ufd1ΔCt* ^*213-342*^ mutant accumulating more damage needing HR for repair than wild-type cells as suggested by the increase in spontaneous Rad22 foci seen in *ufd1ΔCt* ^*213-342*^ cells (Figure 4B). A similar dependency on recombination factors was previously reported for STUbL mutants [26].

The genetic interactions between *ufd1* and a gene central to post-replication repair (PRR), *rhp18*, were more complex. Rhp18 stimulates mono-ubiquitylation of PCNA as part of the PRR response to tolerate replication-stalling lesions encountered during S-phase [21,23]. This mono-ubiquitylation leads to error-prone replication past the lesion by TLS polymerases. PCNA can also be further modified by a different set of enzymes and this stimulates a more error-free mechanism of damage bypass thought to involve a switch in template to the newly synthesized sister strand [21-23,70,71]. We found that the *ufd1ΔCt* ^*213-342*^ mutation was epistatic to *rhp18∆* for growth and HU sensitivity and the double mutant showed a cumulative sensitivity to UV irradiation (Figure 5C). These phenotypes are consistent with Ufd1 and Rhp18 operating in separate pathways. They are consistent with Ufd1 mediating aspects of HR necessary to overcome replication-fork damage such as caused by prolonged HU treatment, and with Rhp18-mediated PRR being primarily required for the tolerance to single-stranded damage, such as photoproducts [23]. Since HR is essential for UV-tolerance when the PRR pathways are inactivated [23], the cumulative sensitivity to UV irradiation in the double *ufd1ΔCt rhp18∆* mutant supports the notion that Ufd1 takes part in HR. The sensitivity of *ufd1ΔCt* ^*213-342*^ cells to CPT however was slightly suppressed by *rhp18∆* (Figure 5B). The *rhp18∆* single mutant is not particularly sensitive to CPT, at least not at the doses tested here (concentrations ≤10µM), consistent with HR being a preferred mode of CPT-induced damage tolerance at these doses. Suppression of the CPT-sensitivity of *ufd1ΔCt* ^*213-342*^ by *rhp18∆* suggests that in addition to performing Rhp18-independent functions, Ufd1 might be necessary either to prevent inappropriate entry into the Rhp18 PRR pathway, or to complete events initiated by Rhp18. To further investigate which branch of PRR is detrimental in the *ufd1ΔCt* ^*213-342*^ background, the epistatic relationship between *ufd1* and a gene controlling entry into the “error-free” pathway of PRR, *ubc13*, was examined. Ubc13 forms part of an E2 heterodimer (Ubc13/Mms2) that stimulates PCNA poly-ubiquitylation, promoting repair by template switching. Interestingly we found that similar to *rhp18∆*, the *ubc13∆* deletion partly suppressed the *ufd1ΔCt* ^*213-342*^ mutant’s sensitivity to CPT (Figure 5C). Thus, entry into the Ubc13-dependent sub-pathway of PRR in response to CPT-induced damage appears deleterious in the absence of functional Ufd1. In response to UV and HU, the same epistatic patterns were observed when combining *ubc13∆* with *ufd1ΔCt* ^*213-342*^ as for *rhp18∆* (data not shown). 

No suppression of the sensitivity to CPT was observed when combining *slx8-1* and *rhp18∆*. Instead a cumulative effect was observed (Figure 5C), suggesting that a function for Ufd1 in controlling Rhp18-mediated responses represents an Slx8-independent role. 

## Discussion

Ufd1 mediates the interactions of the Cdc48/Ufd1/Npl4 complex with ubiquitylated proteins, permitting the extraction of these proteins from higher-order complexes in an energy-driven process catalyzed by the Cdc48 ATPase [38,39]. Once mobilized, the ubiquitylated proteins can either be degraded or allowed to perform new tasks. The specificity of targeting and its outcomes are governed through exchangeable interaction partners of Cdc48/Ufd1/Npl4, including both ubiquitin-binding and -processing co-factors [43,72,73]. Our observations lead us to propose that Ufd1 operates in the STUbL pathway to decide the fate of nuclear proteins sumoylated as part of the DDR. 

**Figure 6:**
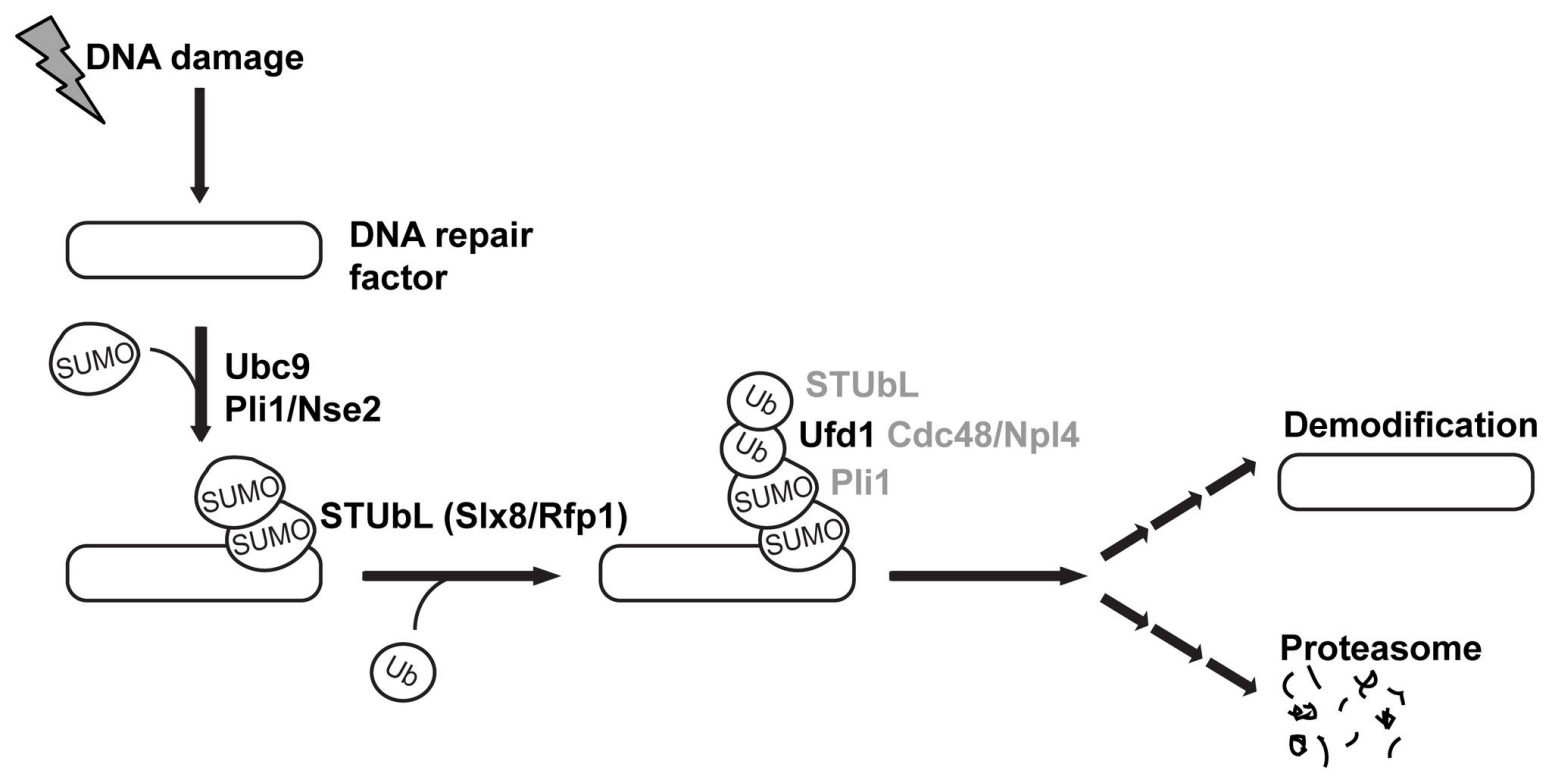
Model depicting a role for Ufd1 (Cdc48/Ufd1/Npl4) in the processing of STUbL substrates. In addition to direct recognition of STUbL modified substrates, interactions between Cdc48/Ufd1/Npl4 and the modifying enzymes (Pli1 and Rfp1/Slx8) could facilitate Cdc48/Ufd1/Npl4 recruitment and a concerted action in the regulation of protein fate.

Both physical and functional overlaps between Ufd1-mediated protein processing and the STUbL pathway were revealed by our experiments. First, a C-terminal domain of Ufd1 bound both the SUMO E3 ligase Pli1 and the STUbL Rfp1 suggesting Cdc48/Ufd1/Npl4 has a function in the turnover of sumoylated proteins (Figure 1A and B). Second, sumoylated species of high molecular weight accumulated in a *ufd1* mutant with a C-terminal truncation of the protein, similar to when STUbL function is compromised (Figure 1E). Given the ability of Ufd1 to mediate the association of the Cdc48/Ufd1/Npl4 complex with ubiquitylated proteins we imagine that Cdc48/Ufd1/Npl4 operates downstream of STUbLs to promote further processing of STUbL substrates and possibly their proteasomal degradation (Figure 6). Third, supporting a model where Ufd1 affects the turnover of SUMO-conjugates in a direct manner, foci formed by Ufd1 in response to DNA damage colocalized with a major SUMO focus at the nuclear periphery (Figure 3C). In the absence of DNA damage, nuclear SUMO foci were more intense in *ufd1ΔCt* ^*213-342*^ cells than in wild type and upon DNA damage the intensity of these foci increased even further in *ufd1ΔCt* ^*213-342*^ cells (Figure 2B). As many proteins are sumoylated during DDR responses [5,7-16] these foci might contain sumoylated repair factors that are normally processed through Ufd1. 

The nature of the physical interactions between Ufd1 and STUbLs is of particular interest. A recent study identified a SIM near the C-terminus of *S. pombe* Ufd1 [74]. This indicated to the authors that Ufd1 might be a STUbL effector since Ufd1 would have the potential of being recruited cooperatively to STUbL substrates co-modified by SUMO and ubiquitin. Here, our observations suggest that Ufd1 might not be solely recruited to STUbL substrates through dual SUMO/ubiquitin recognition of the substrates but by additional interactions between Ufd1 and the modifying enzymes Pli1 and Rfp1. We found that the last 95 aa of Ufd1 are sufficient for these interactions. Mutations in the Ufd1 SIM, which is located at the very C-terminus of Ufd1, abrogated the two-hybrid interactions of Ufd1 with both Pli1 and Rfp1 (Figure S2). This indicates that the interactions are enhanced by SUMO moieties bound to the SIM of Ufd1, or that, in some other way, the same portion of Ufd1 is required for its interactions with SUMO, Pli1 and Rfp1 (Figure S2). Our *in vitro* GST pull-downs suggested that Ufd1 has intrinsic affinity for both Pli1 and Rfp1 (Figure 1B). We also found that the Pli1 SIM is neither necessary nor sufficient for the two-hybrid interaction between Pli1 and Ufd1 in *S. cerevisiae*, supporting the conclusion that the two proteins establish direct contact with each other rather than – or in addition to being bridged by SUMO moieties (Figure S1). According to earlier studies Ufd1 and Cdc48 are sumoylated in *S. cerevisiae* [75,76]. Hannich et al. (2005) [75] also identified a putative SIM in Cdc48 and they proposed that functional interactions between Cdc48 and Ufd1 might be regulated by sumoylation. Interactions between the sumoylation pathway and Cdc48 in *S. cerevisiae* were also detected in other large-scale studies [77,78]. Collectively, these and our observations lead to the view that a combination of direct interactions and indirect associations through SUMO and ubiquitin modulate the formation of higher-order complexes comprising Cdc48/Ufd1/Npl4, Pli1, STUbLs and their substrates to enable a concerted action in the regulation of protein fate. 

The slow growth and genome instability of STUbL mutants have been attributed to an accumulation of one or several poly-sumoylated species since deleting Pli1, overexpressing the desumoylase Ulp2, or preventing chain formation by mutating major lysine acceptor sites in SUMO suppresses these phenotypes [26,27,31,74]. The observed genome instability of the *ufd1ΔCt* ^*213-342*^ mutant is unlikely to be explained solely by an accumulation of the same SUMO conjugates as deleting Pli1 did not suppress the *ufd1ΔCt* ^*213-342*^ mutation. This is consistent with the emerging picture that Cdc48/Ufd1/Npl4 is required for several aspects of genome maintenance [44-54]. 

Recent studies have proposed several functions for Cdc48/Ufd1/Npl4 in ubiquitin-mediated maintenance of genome integrity. In metazoans, Cdc48/Ufd1/Npl4 deficient cells fail to degrade the replication-licensing factor Cdt1 and arrest in S-phase with reduced DNA content and Rad51 foci [46-49]. Another proposed function for Cdc48/Ufd1/Npl4, in mammalian cells, is in the response to DSBs where ubiquitin-dependent recruitment of Cdc48/Ufd1/Npl4 permits the removal of K48-linked ubiquitylated species from the sites of lesion and the recruitment of downstream repair factors including 53BP1 [51]. Mechanistically, recruitment of 53BP1 is believed to depend on Cdc48/p97 dissociating the L3MBTL1 polycomb group protein from histone H4K20me2, a binding site common to both 53BP1 and L3MBTL1 [52]. Although this mechanism might not be conserved in yeast as no homolog of L3MBTL1 has been found, the *S. pombe ufd1ΔCt* ^213-342^ mutant was clearly defective in some aspects of HR. The *ufd1ΔCt* ^*213-342*^ mutant was sensitive to DSB-inducing agents; *ufd1ΔCt* ^*213-342*^ cells treated with Zeocin arrested with bright Rad22 foci; and Rad22 accumulated in high-molecular-weight forms in *ufd1ΔCt* ^*213-342*^ cells (Figure 4F). Hence, our observations permit to add Rad22 to the short list of known factors whose processing depends on Cdc48/Ufd1/Npl4. Evidence for *S. cerevisae* Rad52 also being a target of Cdc48-Ufd1-Npl4 was recently provided by Bergink et al. [79]. These authors proposed that Cdc48-Ufd1-Npl4 recognizes sumoylated Rad52 through the Ufd1 SIM and mediates the disassociation of Rad52 and Rad51 from each other and from DNA. Whether this specific pathway involves STUbL activity was not investigated. Even though the study indicates that sumoylation is sufficient for the association of Cdc48-Ufd1-Npl4 with Rad52, STUbL-mediated ubiquitylation of Rad52 might strengthen this interaction *in vivo* or alternatively operate downstream to regulate Rad52 fate. Consistent with this proposition, *S. cerevisae* Rad52 is an *in vitro* STUbL substrate [80,81]. 

PRR mediates tolerance to DNA damage encountered during S-phase [71]. Entry into the PRR pathway is controlled by the ubiquitin E3 ligase Rhp18. Rhp18-mediated mono-ubiquitylation of PCNA [21,23] stimulates translesion synthesis by recruiting TLS polymerases able to replicate across fork-stalling lesions [22,82,83]. Further elongation of the mono-ubiquitin tag on PCNA by Ubc13/Mms2 stimulates an error-free mode of damage bypass known as template switching [21,70,84]. Here we observed that deleting *rhp18* or *ubc13* partially suppressed the CPT sensitivity of *ufd1ΔCt* ^*213-342*^ cells (Figure 5C). These data indicate that entry into the template switch branch of PRR is detrimental in the *ufd1ΔCt* ^*213-342*^ background, at least when dealing with CPT-induced damage. The residual CPT-sensitivity of the *ufd1ΔCt* ^*213-342*^ *rhp18∆* double mutant indicates additional role(s) for Ufd1 in repair of CPT-induced damage. Also consistent with Ufd1 and Rhp18 acting separately, the *ufd1ΔCt* ^*213-342*^ *rhp18∆* double mutant showed synergistic sensitivity to UV irradiation, which creates damage that critically depend on HR when PRR is compromised [23].

Our observations strengthen and refine the notion that Cdc48-Ufd1-Npl4 is essential to the maintenance of genome integrity by acting in several pathways including the STUbL pathway. We propose that Ufd1 acts downstream and/or in concert with SUMO ligases and STUbLs to remove or recycle sumoylated species. Given the widespread use of sumoylation in repair complexes this action might be necessary to the dynamics of repair at multiple points in the DDR. A broader relationship between sumoylation and the Cdc48-Ufd1-Npl4 complex might affect cellular processes other than the DDR.

## Materials and Methods

### Strains and media

Genotypes are listed in Table S1. *S. pombe* strains were propagated in yeast extract medium or in Edinburgh minimal medium (EMM2) with indicated supplements. *S. cerevisiae* was propagated in YPD or SC drop-out media.

### Two-hybrid screens

The Pli1 and Rfp1 ORFs were cloned into pGBKT7 (Clontech) as in-frame fusions with the ORF for the Gal4 DNA-binding domain (GBD) and each plasmid was transformed into PJ69-4A (*MATa trp1-901 leu2-3,112 ura3-52 his3-200 gal4Δ gal80Δ LYS2::GAL1-HIS3 GAL2-ADE2 met2::GAL7-lacZ*; [85]), selecting for Trp^+^. The resulting strains were transformed with a two-hybrid library in pGAD-GH in which *S. pombe* cDNAs were fused to the sequence coding for the Gal4 transcription-activating domain (Clontech). More than 5 million Leu^+^ Trp^+^ transformants were obtained for Rfp1 and more than 10 million for Pli1. Separate selections were applied for activation of the *ADE2* reporter gene (by plating on SC-leu-trp-ade) and for the activation of the *HIS3* reporter gene (by plating on SC-leu-trp-his+3 mM 3-aminotriazol, or 1.5 mM 3-aminotriazol). Ade^+^ transformants were subsequently tested for expression of *HIS3* and, *vice versa*, His^+^ transformants were tested for the expression of *ADE2*. Plasmids were extracted from Ade^+^ His^+^ candidates, retested by co-transformation with bait-encoding plasmids or empty vectors, and partially sequenced. The clones presented in Figure 1 represent only a fraction of the clones capable of activating both the *ADE2* and *HIS3* reporter genes in a bait-dependent manner identified in the screens.

### Construction of *ufd1∆Ct* ^213-342^ strains

To produce Ufd1 lacking amino acids 213-343, a plasmid was prepared as follows: part of the *ufd1* ORF (nucleotide 26-969) was amplified by PCR on *S. pombe* genomic DNA using as primers GTO-336 (CCATCCCGGGGTACGTTGACTTACTCACGTATTAG, introducing an *Xma*I site, underlined) and GTO-337 (GTATGGCGCGCCTCATAACCGATGGGGGGGATCAAAATC, introducing an *Asc*I site, underlined). GTO-337 was designed with an extra G in a row of six guanines, which upon genomic integration of the amplified sequence should create a single nucleotide insertion in the *ufd1* ORF at nucleotide 953 causing a frameshift and a resulting stop at codon 213. *ufd1* 3’flanking DNA (933 bp downstream of the ORF) was amplified with GTO-338 (CCATGAGCTCTGTTAATCGTCTCAAGTTATTACTTG, introducing a *Sac*I site, underlined) and GTO-339 (GTCTACTAGTGAGGAGCTTGACGGCGTCTGCGAGG, introducing an *Spe*I site, underlined). Coding and 3’fragments were cloned into pFA6a-hphMX6 [86] using the restriction sites introduced in the primers and the resulting plasmid was digested with *Xma*I-*Spe*I before transformation into a diploid strain, selecting for hygromycin-resistance. *ufd1∆Ct* ^213-342^ hygromycin-resistant haploid progeny were obtained by tetrad dissection of diploid JK5. Correct integration of the construct was confirmed by PCR analysis using the primers GTO-336 (see above) and GTO-340 (GGGCAGCGTTCTTAGCACGAGCTTC) or GTO-336 and GTO-342 (CGCTATACTGCTGTCGATTCG) and by Southern blotting. The *hph1* gene was subsequently replaced with the nourseothricin resistance gene (*nat1*) by transformation of the *ufd1∆Ct* ^213-342^ strain JK9 with the pCR2.1-nat plasmid digested with EcoRI [86].

### GST pull-downs

Pli1 and Rfp1 were expressed as GST-fusions from the pGEX-KG vector in the bacterial strain BL21 (DE3) pLysS cells and purified under native conditions (1x PBS, 0.1% Triton, 1 mM DTT, 1x Complete protease inhibitor Cocktail EDTA-free from Roche) on a glutathione–Sepharose column (Amersham) using standard protocols. For ^35^S-methionine-labeling of Ufd1, a PCR product amplified from genomic DNA with the primers GTO-483 (GCATGGATCCATGATGACTACACTTAGCCTTGAGCC, introducing a *Bam*HI site, underlined) and GTO-484 (CGATGAATTCTTAAGCATCAATATCGATTGGGTC, introducing an *Eco*RI site, underlined) was cloned into pING14 [87] and used to produce a C-terminal fragment corresponding to amino acids 110-342 of Ufd1 by *in vitro* translation using the the SP6 TNT-coupled reticulocyte lysate kit (Promega) and ^35^S-methionine (Perkin Elmer). ~20 µg of GST or GST-fusion proteins were incubated with the *in vitro* translation product overnight at 4πC in 1x PBS, 0.1% NP-40, 5% glycerol, 1 mM DTT. Glutathione–Sepharose beads were added for 3 hr and washed 5 times. Bound proteins were released by boiling in 1x Laemmli sample buffer. Released proteins were resolved by gel electrophoresis and Ufd1 was detected by phosphorimaging*.*

### Anti-SUMO Western blotting

Whole-cell extracts for immunoblot analysis of total SUMO species were prepared from cells growing exponentially in supplemented EMM2. Cells were lysed in TNET buffer (200 mM NaCl, 0.1% Triton, 0.01% SDS, 50 mM Tris-HCl pH8.0, 50 mM EDTA, 10 mM N-ethylmaleimide, 1x Complete protease inhibitor Cocktail EDTA-free from Roche) with glass beads in a Fastprep® Instrument kept at 4 °C. Equal amounts of protein extracts boiled in 1x Laemmli sample buffer were separated on a 4-20% Tris-glycine gradient gel (Lonza), transferred to a nitrocellulose/MCE membrane (Advantec), and probed with an anti-SUMO rabbit antibody ([4]; kindly provided by J. Seeler) followed by a horseradish peroxidase (HRP)-conjugated swine anti-rabbit IgG secondary antibody (Dako). Detection was with an ECL plus kit (GE Healthcare). 

### GFP immunoprecipitations

For immunoprecipitations of Rad22-YFP, 300 ml of cultures growing exponentially in YEL were harvested at OD_600_~0.4, resuspended in 800 µl lysis buffer (10 mM Tris pH 7.5, 150 mM NaCl, 0.5 mM EDTA, 10% glycerol, 0.5% NP40, 10 mM N-ethylmaleimide, 1 mM PMSF, 1x Complete protease inhibitor Cocktail EDTA-free from Roche, 1µg/µl DNaseI and 2.5 mM MgCl_2_) and lyzed by the glass bead method. Protein concentrations were determined using a Nanodrop Spectrophotometer (280 nm). Equal amounts of total protein (~54 mg per sample) were diluted 2x in binding buffer (same as lysis buffer but without NP-40, DNaseI and MgCl_2_) to a final volume of ~1.5 ml and incubated with 30 µl pre-equilibrated GFP-Trap®_M beads (Chromotek) for 3 hr at 4°C under constant mixing. Beads were washed 3 times in binding buffer. Bound proteins were released by incubation at 90°C for 10 min in 2x Laemmli buffer, separated on a 4-20% Tris-glycine gradient gel (Lonza), and transferred to a nitrocellulose/MCE membrane (Advantec). GFP-trapped proteins were detected using an anti-GFP mouse antibody (Roche) in combination with (HRP)-conjugated goat anti-mouse from Thermo Scientific and an ECL plus kit (GE Healthcare).

For Cdc48-GFP co-IP experiment, 200 ml of cultures were grown in YEL to an OD_600_~0.9. Cells were lysed in 1.4 ml lysis buffer (25 mM Tris pH 7.5, 50 mM NaCl, 10% glycerol, 0.1% Triton 100-X, 2 mM DTT, 1 mM PMSF and protease inhibitors) with glass beads and ~60 mg of total protein per sample was mixed with ~130 µl pre-equilibrated GFP-Trap®_M beads (Chromotek) for 1-2 hr at 4°C. Beads were washed 4 times with an increased salt concentration (300 mM NaCl) before elution of bound material by boiling in Laemmli buffer. Cdc48-GFP was identified by the anti-GFP mouse antibody from Roche and co-precipitated Slx8-Myc was identified with a Myc-tag mouse antibody from Cell Signaling Technology. 

### Fluorescence microscopy

Strains used for fluorescence microscopy were propagated overnight at 30°C in supplemented EMM2, to early exponential phase. Rad22-YFP was expressed as a replacement of the *rad22* ORF [88]. Ufd1-YFP was expressed from the *nmt1* promoter [61] in cells with a full deletion of the endogenous *ufd1* gene. The *nmt1-ufd1-YFP* construct integrated at *leu1* fully complemented the full deletion of *ufd1* in media lacking thiamine in which the *nmt1* promoter is active. For treatment with Zeocin, cells were incubated with 350 µg/ml Zeocin (Invitrogen) for 1-5 hr prior to microscopy as indicated. For the Zeocin-recovery experiment, cells were pelleted after a 1 hr treatment with 350 µg/ml Zeocin and they were allowed to grow in fresh supplemented EMM2 medium for ~13 hr in a shaking 30°C incubator. Images were obtained with a Zeiss Axio Imager Z.I (Carl Zeiss) microscope linked to an Orca-ER CCD camera (Hamamatsu). Images were analyzed using Volocity (version 5, Improvision).

### Quantification of GFP-SUMO focus intensity

The intensity of the brightest GFP-SUMO focus was measured in wild-type and *ufd1ΔCt* ^*213-342*^ cells before and after Zeocin treatment using the Volocity analysis module (version 5, Improvision). The Magic Wand ROI tool was used to select entire nuclei in 3-D. A value for the voxel of highest intensity was obtained for each nucleus. Three independent experiments were performed, producing very similar results. Values obtained in one experiment were compiled to prepare the graphs shown in Figure 2. 

### RNA extraction and real-time PCR

Cells were propagated in YEL medium to an OD600~ 0.2 and RNA was extracted as described in Lyne et al., 2003 [89].  Three independent cultures were processed for each strain. The following primers were used for transcript detection by real-time PCR: *rad22* ^*+*^, GTO-551 (5’ GACAATCAAAGATGGTGCCTATC 3’) and GTO-552 (5’ CATCTGTAGTGCCCTCTTTCTTG 3’); *act1* ^*+*^, TJO-55 (5’ CTGTTTTGTCTTTGTATGCC 3’) and TJO-58 (5’ TAAGGTAGTCAGTCAAGTCA 3’). Real-time PCR was performed on a BioRad CFX96 system, using a QuantiTect SYBR Green PCR Kit from Qiagen according to the manufacturer instructions.  The reverse-transcription step was performed at 50°C for 30 min.   Following reverse transcription, the samples were heated at 95°C for 15 min, and subjected to 39 cycles of (95°C for 15 s, 55°C for 30 s and 72°C for 30 s).  All reactions were set up in triplicates and the melting curve of all PCR products was determined after amplification.  Ten-fold dilution series of genomic DNA were used to determine primer efficiencies and the exponential range of amplification for each primer pair. Mean normalized expression (MNE) values for transcript levels were calculated according to the equation MNE= (E_ref_)^CTref,mean^  /  (E_target_)^CTtarget,mean^ [90].         

## Supporting Information

Figure S1 **Interactions of Pli1 subclones with Ufd1 and SUMO.** (A) Schematic representation of Pli1 domains and Pli1 subclones used for the two-hybrid assays displayed in (B). (B) Yeast two-hybrid interactions. 10-fold dilution series of S. *cerevisae* strain PJ69-4A expressing the indicated fusion to GAD or GBP were spotted on the indicated media. GAD-Ufd1 expresses the C-terminal domain of Ufd1 (aa 248-342) fused to the Gal4 DNA activation domain (GAD). GAD-Pmt3 expresses *S. pombe* SUMO fused to GAD. Protein interactions result in activation of the ADE2 and HIS3 reporter genes in the tester strain and growth on SC-leu-trp-ade and SC-leu-trp-his+3 mM3-AT. Blue arrows point to Pli1 subclones that do not interact with SUMO, yet interact with Ufd1. The red arrow points to a Pli1 subclone interacting strongly with SUMO but not Ufd1. Globally, the interactions of Pli1 with SUMO require the Pli1 SIM or SP-RING domain whereas the interactions of Pli1 with Ufd1 occur in the absence of these two domains.
(TIF)

Figure S2 **Mutations in the Ufd1 SIM abrogate the two-hybrid interactions of Ufd1 with Pli1 and Rfp1.** (A) Representation of the Ufd1 C-terminal portion used for two-hybrid assays, annotating a SIM motif in the last seven amino acids that was either deleted (Ufd1Ct∆SIM) or mutated (Ufd1CtDAADADA) for the interaction tests shown below. (B) Transformants of *S. cerevisiae* strain PJ69-4A expressing the indicated GAD- and GBD-fusion proteins were spotted onto selective media to test for interactions.
(TIF)

Figure S3 **Supplemental data to Figure 4.** (A) Fluorescence imaging of Rad22-YFP in wild type and *ufd1ΔCt* ^*213-342*^ mutant before and after treatment with Zeocin (300 µg/ml for 1 hr.) (B) *rad22* transcript levels are similar in wild-type and *ufd1ΔCt* ^*213-342*^ mutant cells. Real-time RT-PCR analysis of *rad22* RNA isolated from wild-type or *ufd1ΔCt* ^*213-342*^ cells. *rad22* RNA quantities were measured relative to actin. Error bars indicate the standard deviation obtained from three independent biological isolates.
(EPS)

Table S1 **Strain Table.**
(DOC)

Table S2 **Number of Rad22-YFP foci and nuclei counted to produce the bar graphs shown in Figure 4C.** Numbers were combined from three independent experiments.
(DOCX)
