# Supplementary figures and images for: Concerted Action of the Ubiquitin-Fusion Degradation Protein 1 (Ufd1) and Sumo-Targeted Ubiquitin Ligases (STUbLs) in the DNA-Damage Response
Source: PLoS One. 2013 Nov 12;8(11):e80442. doi: 10.1371/journal.pone.0080442 (PMC3827193; doi:10.1371/journal.pone.0080442)

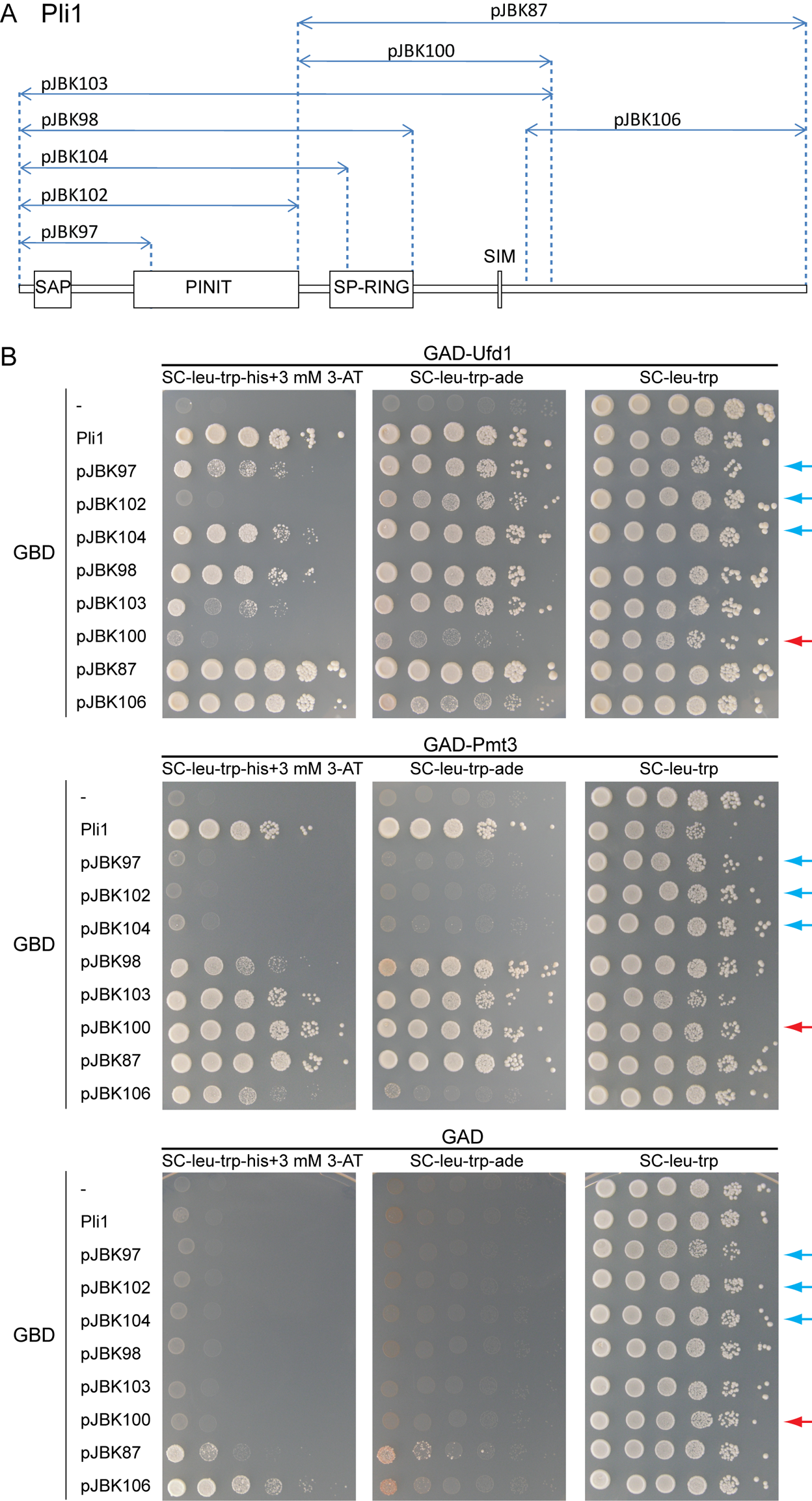

Supplement: Figure S1 — Interactions of Pli1 subclones with Ufd1 and SUMO. (A) Schematic representation of Pli1 domains and Pli1 subclones used for the two-hybrid assays displayed in (B). (B) Yeast two-hybrid interactions. 10-fold dilution series of S. cerevisae strain PJ69-4A expressing the indicated fusion to GAD or GBP were spotted on the indicated media. GAD-Ufd1 expresses the C-terminal domain of Ufd1 (aa 248-342) fused to the Gal4 DNA activation domain (GAD). GAD-Pmt3 expresses S. pombe SUMO fused to GAD. Protein interactions result in activation of the ADE2 and HIS3 reporter genes in the tester strain and growth on SC-leu-trp-ade and SC-leu-trp-his+3 mM3-AT. Blue arrows point to Pli1 subclones that do not interact with SUMO, yet interact with Ufd1. The red arrow points to a Pli1 subclone interacting strongly with SUMO but not Ufd1. Globally, the interactions of Pli1 with SUMO require the Pli1 SIM or SP-RING domain whereas the interactions of Pli1 with Ufd1 occur in the absence of these two domains. (TIF) [file pone.0080442.s001.tif]

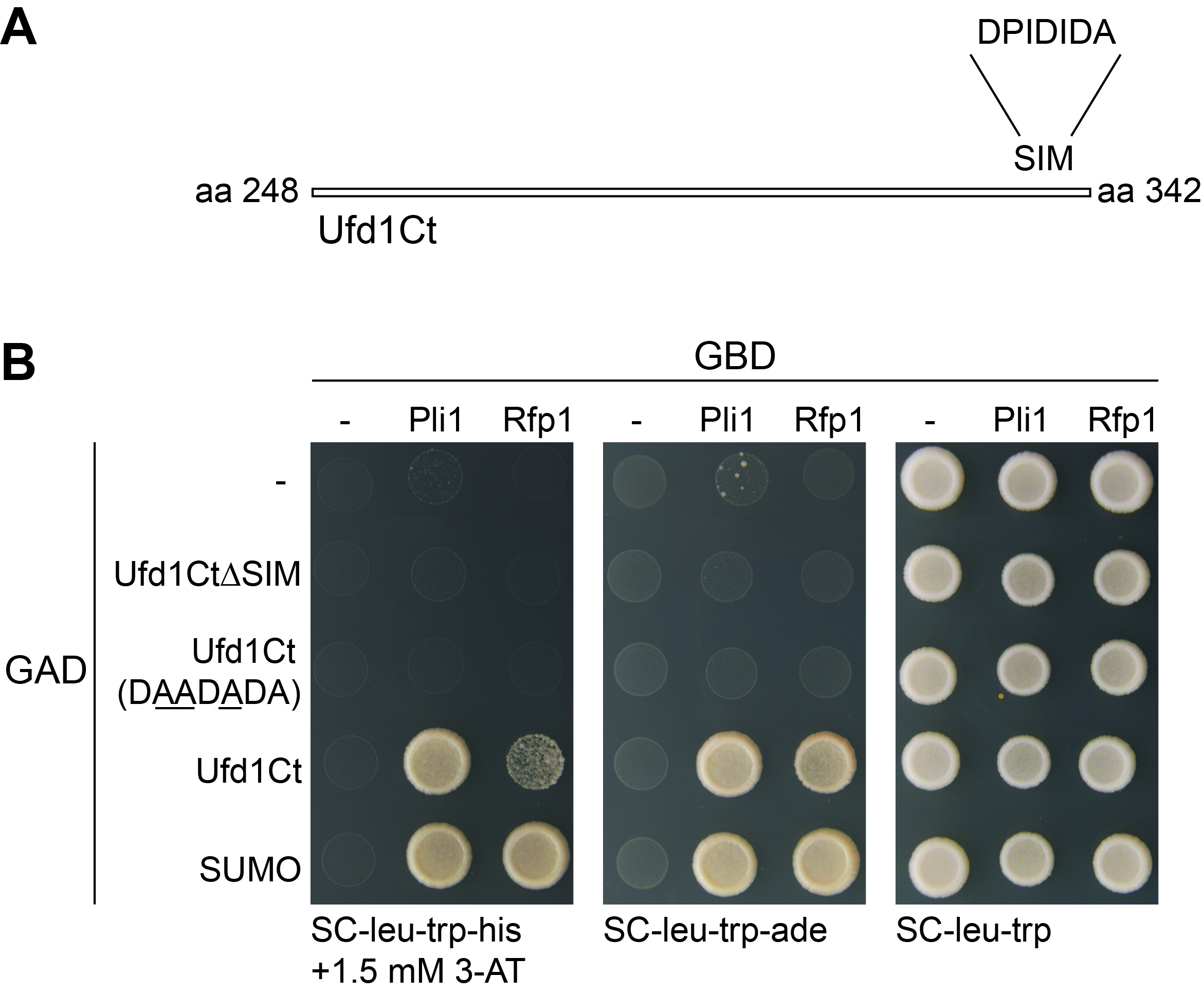

Supplement: Figure S2 — Mutations in the Ufd1 SIM abrogate the two-hybrid interactions of Ufd1 with Pli1 and Rfp1. (A) Representation of the Ufd1 C-terminal portion used for two-hybrid assays, annotating a SIM motif in the last seven amino acids that was either deleted (Ufd1Ct∆SIM) or mutated (Ufd1CtDAADADA) for the interaction tests shown below. (B) Transformants of S. cerevisiae strain PJ69-4A expressing the indicated GAD- and GBD-fusion proteins were spotted onto selective media to test for interactions. (TIF) [file pone.0080442.s002.tif]
