# Supplementary material for: Concerted Action of the Ubiquitin-Fusion Degradation Protein 1 (Ufd1) and Sumo-Targeted Ubiquitin Ligases (STUbLs) in the DNA-Damage Response
Source: PLoS One. 2013 Nov 12;8(11):e80442. doi: 10.1371/journal.pone.0080442 (PMC3827193; doi:10.1371/journal.pone.0080442)
Supplement: Table S1 — Strain Table. (DOC) [file pone.0080442.s004.doc]

**Table S1**

| Strain | Genotype | Study |
| --- | --- | --- |
| ∆Sph2 | *mat1-Msmt-0 leu1-32 ura4-D18 ade-210 pli1Δ::kanMX6* | Xhemalce  *et al.* 2007 [91] |
| JK8 | *h^+^  leu1-32 ura4-DS/E ade-210 arg-* | This study |
| JK9 | *h^90^  leu1-32 ura4-DS/E ade-216 imr1R(NcoI)::ura4^+^ori1*  *ufd1ΔCt^213-342^::hphMX6* | This study |
| JK10 | *h^+^  leu1-32 ura4-DS/E ade-216 imr1R(NcoI)::ura4^+^ori1*  *ufd1ΔCt^213-342^::hphMX6* | This study |
| JK11 | *h^90^  leu1-32 ura4-DS/E ade-210 arg-* | This study |
| JK44 | *mat1-Msmt-0 leu1-32 ura4-D18 ade-210 imr1R(NcoI)::ura4^+^ori1* | This study |
| JK45 | *h^90^  leu1-32 ura4-DS/E ade-216 pli1Δ::kanMX6*  *ufd1ΔCt^213-342^::hphMX6* | This study |
| JK46 | *mat1-Msmt-0 leu1-32 ura4-DS/E ade-210 imr1R(NcoI)::ura4^+^ori1* *pli1Δ::kanMX6* | This study |
| JK47 | *h^90^  leu1-32 ura4-D18 ade-216 ufd1ΔCt^213-342^::hphMX6* | This study |
| JK60 | *h^90^  leu1-32 ura4-DS/E ade-216 imr1R(NcoI)::ura4^+^ori1*  *ufd1ΔCt^213-342^::natMX6* | This study |
| JK82 | *h^90^  leu1-32 ura4-DS/E ade-210 ufd1ΔCt^213-342^::natMX6* | This study |
| JK87 | *mat1-Msmt-0* *leu1-32 ura4-DS/E ade-216*  *ufd1ΔCt^213-342^::natMX6 rad22-YFP-kan* | This study |
| JK88 | *mat1-Msmt-0 leu1-32 ura4-DS/E imr1R(NcoI)::ura4^+^ori1*  *ufd1ΔCt^213-342^::natMX6 rad22-YFP-kan* | This study |
| JK90 | *h^-^  leu1-32 ura4-DS/E ade-210 ufd1ΔCt^213-342^::natMX6 rhp18Δ::ura4* | This study |
| JK91 | *h^-^  leu1-32 ura4-DS/E ade-210 arg- rhp18Δ::ura4* | This study |
| JK92 | *h^90^  leu1-32 ura4-DS/E ade-216 ufd1ΔCt^213-342^::natMX6* | This study |
| JK93 | *h^90^  leu1-32 ura4-DS/E ade-216 arg-* | This study |
| JK95 | *h^90^  leu1-32 ura4-DS/E ade-210 ufd1ΔCt^213-342^::natMX6 rhp18Δ::ura4* | This study |
| JK97 | *h^90^  leu1-32 ura4-DS/E ade-210 ufd1ΔCt^213-342^::natMX6 rhp18Δ::ura4* | This study |
| JK98 | *h^90^  leu1-32 ura4-DS/E ade-216 slx8-1:myc:kanMX6*  *ufd1ΔCt^213-342^::natMX6* | This study |
| JK109 | *h^+^  ade-210 his3-1 ufd1ΔCt^213-342^::natMX6 rhp51::his3* | This study |
| JK110 | *h^90^  leu1-32 ade-216 his3-1 ufd1ΔCt^213-342^::natMX6* | This study |
| JK111 | *h^90^  leu1-32 ura4-DS/E ade-210 his3-1* | This study |
| JK112 | *h^+^  ura4-DS/E ade-216 his3-1 rhp51::his3* | This study |
| JK113 | *h^-^  leu1-32 ura4-DS/E ade-216 ufd1ΔCt^213-342^::natMX6* | This study |
| JK114 | *h^90^  leu1-32 ura4-DS/E ade-210 arg-* | This study |
| JK115 | *h^90^  leu1-32 ura4-DS/E ade-210 arg- rqh1Δ::ura4* | This study |
| JK116 | *h^-^  leu1-32 ura4-DS/E ade-216 ufd1ΔCt^213-342^::natMX6 rqh1Δ::ura4* | This study |
| JK124 | *h^90^  leu1-32 ura4-D18 ade-210/216 pli1Δ::kanMX6*  *ufd1ΔCt^213-342^::hphMX6* | This study |
| JK295 | *h^90^  leu1-32 ura4 slx8:myc:: natMX6* | This study |
| JK296 | *h^-^  leu1-32 ura4-D18 slx8-1:myc:kanMX6 rhp18Δ::ura4* | This study |
| JK298 | *h^90^  leu1-32 ura4-D18 slx8-1:myc:kanMX6 rhp18Δ::ura4* | This study |
| JK299 | *h^90^ leu1-32 ura4-D18 ade-DN/N slx8-1:myc::kanMX6 rhp18Δ::ura4* | This study |
| JK309 | *h^90^  leu1-32 ura4 slx8:myc:: natMX6 cdc48-GFP:kanR* | This study |
| JK332 | *h^90^ ura4-DS/E ade-216 arg- leu1::ufd1-YFP-FLAG2-His6 ufd1Δ::hphMX6* | This study |
| JK333 | *h^90^  ura4-DS/E ade-210 arg- leu1::ufd1-YFP-FLAG2-His6 ufd1Δ::hphMX6* | This study |
| JK334 | *h^90^  leu1-32 ura4-D18 ade-704 ufd1ΔCt^213-342^::natMX6*  *pmt3:pYC11(Leu+)-GFP-pmt3* | This study |
| JK336 | *h^90^  leu1-32 ura4-D18 ade-704 pmt3:pYC11(Leu+)-GFP-pmt3* | This study |
| JK339 | *h^90^  ura4-DS/E ade-210 arg- pmt3:nat-CFP-pmt3*  *leu1+::ufd1-YFP-FLAG2-His6 ufd1Δ::hphMX6* | This study |
| JK346 | *h^90^  leu1-32 ura4 ade6 ufd1ΔCt^213-342^::natMX6 ubc13Δ:: ura4* | This study |
| JK347 | *h^90^  leu1-32 ura4 ade6 ufd1ΔCt^213-342^::natMX6 ubc13Δ:: ura4* | This study |
| JK349 | *h^90^  leu1-32 ura4 ade6 ubc13Δ:: ura4* | This study |
| JK380 | *mat1-Msmt-0 leu1-32 ura4-DS/E ade-216 ufd1ΔCt^213-342^::natMX6* | This study |
| FY17243 | *h^-^  leu1-32 cdc48-GFP:kanR* | M. Yanagida |
| NBY1008 | *h^-^  leu1-32 ura4-D18 slx8-1:myc:kanMX6* | Prudden  *et al.* 2007 [26] |
| PG1636 | *h^90^  leu1-32 ura4-DS/E ade-210 arg-* | Thon *et al*. 2002 [92] |
| PG1637 | *h^90^  leu1-32 ura4-DS/E ade-216 arg-* | Thon *et al.* 2002 [92] |
| PG3453 | *mat1-Msmt-0 leu1-32 ura4-DS/E ade-216* | This study |
| PI131 | *h^90^  mat3-M(EcoRV)::ura4 leu1-32 ura4-D18 ade-216 mts3-1* | Nielsen *et al.* 2002 [93] |
| Y1928 | *mat1-Msmt-0* *leu1-32 ura4-D18 rad22-YFP-kan* | Noguchi  *et al.* 2007 [88] |
