# Supplementary material for: Concerted Action of the Ubiquitin-Fusion Degradation Protein 1 (Ufd1) and Sumo-Targeted Ubiquitin Ligases (STUbLs) in the DNA-Damage Response
Source: PLoS One. 2013 Nov 12;8(11):e80442. doi: 10.1371/journal.pone.0080442 (PMC3827193; doi:10.1371/journal.pone.0080442)
Supplement: Table S2 — Number of Rad22-YFP foci and nuclei counted to produce the bar graphs shown in Figure 4C. Numbers were combined from three independent experiments. (DOCX) [file pone.0080442.s005.docx]

| **Table S2** | **Wild type** | | ***ufd1∆Ct^213-342^*** | |
| --- | --- | --- | --- | --- |
| **Cell cycle stage** | Number of foci | Total nuclei | Number of foci | Total nuclei |
| S | 18 | 182 | 41 | 200 |
| S/G2 | 33 | 111 | 60 | 187 |
| G2 | 42 | 458 | 127 | 559 |
| M/G1 | 0 | 62 | 2 | 36 |
| Cells with 2 or more septa | 1 | 8 | 9 | 44 |
| **Total all phases** | **94** | **821** | **239** | **1026** |
